# Non-Opioid Peptides Targeting Opioid Effects

**DOI:** 10.3390/ijms222413619

**Published:** 2021-12-19

**Authors:** Katarzyna Kaczyńska, Piotr Wojciechowski

**Affiliations:** Department of Respiration Physiology, Mossakowski Medical Research Institute, Polish Academy of Sciences, Pawińskiego 5 St., 02-106 Warsaw, Poland; pwojciechowski@imdik.pan.pl

**Keywords:** non-opioid peptides, opioid side effects, antinociception, tolerance, respiratory depression

## Abstract

Opioids are the most potent widely used analgesics, primarily, but not exclusively, in palliative care. However, they are associated with numerous side effects, such as tolerance, addiction, respiratory depression, and cardiovascular events. This, in turn, can result in their overuse in cases of addiction, the need for dose escalation in cases of developing tolerance, and the emergence of dose-related opioid toxicity, resulting in respiratory depression or cardiovascular problems that can even lead to unintentional death. Therefore, a very important challenge for researchers is to look for ways to counteract the side effects of opioids. The use of peptides and their related compounds, which have been shown to modulate the effects of opioids, may provide such an opportunity. This short review is a compendium of knowledge about the most important and recent findings regarding selected peptides and their modulatory effects on various opioid actions, including cardiovascular and respiratory responses. In addition to the peptides more commonly reported in the literature in the context of their pro- and/or anti-opioid activity—such as neuropeptide FF (NPFF), cholecystokinin (CCK), and melanocyte inhibiting factor (MIF)—we also included in the review nociceptin/orphanin (N/OFQ), ghrelin, oxytocin, endothelin, and venom peptides.

## 1. Introduction

Opioids are not only potent painkillers with anti-inflammatory properties [1,2,3] but also share a variety of additional effects that may limit the provision of effective analgesia. The most important adverse effects are addiction [2,4] and frequent respiratory depression that can potentially be fatal [5,6]. Thus, opioid usage exposes patients to the development of addiction, the emergence of tolerance, and also unnecessary pain while using inadequate opioid dosing due to fear of respiratory depression. Endogenous (dynorphins, endorphins, enkephalins) and exogenous (morphine) opioids act at the three opioid receptors, mu (μOR), delta (δOR), and kappa (κOR) [7]. However, opioid activity can be regulated not only by direct action pointed at these receptors [8], but also by a number of active peptides which do not present affinity or any direct action toward them. It is extremely important to look for ways to reduce the side effects of opioid therapy, and one way might be to consider peptides that reduce or enhance opioid effects.

The repertoire of various peptides that fall into the category of compounds that affect opioid properties include neuropeptide FF (NPFF), cholecystokinin (CCK), melanocyte inhibiting factor (MIF) [9,10], nociceptin/orphanin (N/OFQ) [11] and several other peptides such as ghrelin, oxytocin, and endothelin A [12]. Some of them, such as NPFF, CCK or MIF, present a dual nature and may show anti-opioid properties, but also act in a similar way to opioids; this all depends, among other factors, on the selective activation of peptide receptors, concentration, or the central or peripheral site of peptide action [9].

The majority of the available data on the effects of various peptides on opioid-related activity are focused on the modulation of antinociceptive responses, tolerance, dependence, or modulation of the reward system. Much fewer data are obtainable on how those peptides affect opioid-induced cardiovascular and respiratory effects, yet the respiratory depression and cardiovascular consequences of opioid use are no less important. It is well known that acute opioid treatment may result in hypotension, orthostasis, and bradycardia, while chronic opioid use or withdrawal can be associated with complications including vascular and arrhythmic sequelae [13,14].

This brief review attempts to summarize the most important and recent findings regarding the pharmacologically confirmed modulatory effects of several endogenous and exogenous peptides on various opioid responses. Particular emphasis is placed on a literature search for the effects of the described peptides on post-opioid respiratory and cardiovascular responses. We have also included information on peptides rarely described in review papers in the context of their pro- and anti-opioid activity, such as ghrelin, oxytocin, endothelin, and venom peptides (Table 1).

## 2. Neuropeptide FF (NPFF)

Neuropeptide FF is a mammalian amidated neuropeptide, isolated in 1985 and described as a pain modulating peptide carrying anti-opioid activity towards morphine-induced analgesia [38,39]. Neuropeptide FF and its two receptors, NPFFR1 and NPFFR2, are involved in numerous activities, such as modulation of opioid-induced tolerance and dependence, anxiety control, drug rewards, pain regulation, and blood pressure regulation [40].

NPFF receptors are high affinity G-protein coupled receptors. Brain neurons with strong NPFFR1 expression were observed mostly in hypothalamic areas linked to neuroendocrine function, while NPFFR2 expressing neurons were present mainly in several subnuclei of the thalamus involved in somatosensory pathways [41] and in the rat spinal cord [42].

The most explored activities of NPFF are modulation of pain and opioid-induced analgesia, which have been previously described in detail by [16,17]. To summarize, NPFF has been delineated to have two different effects on pain perception, depending on the site of application. Intrathecal (it) administration of NPFF produced antinociceptive activity by provoking the analgesia and potentiation of opioid effects [43]. Conversely, supraspinal or intracerebroventricular (icv) treatment revealed the anti-opioid face of this peptide and presented pro-nociceptive effects, reversing morphine-induced analgesia [44]. Pain modulation was not related to opioid receptors, and NPFF receptors appear to be key players [15]. The pronociceptive effect is thought to depend primarily on the activation of the NPFFR1 subtype and the antinociceptive effect on the NPFFR2 subtype [19]. However, in the absence of a selective antagonist that distinguishes the effects of subtype receptor activation, this has not been conclusively supported. Studies on mice over-expressing NPFFR2 showed that they are more sensitive to mechanical and thermal noxious stimuli, while acute morphine antinociception was not changed in comparison to wild type littermates. However, transgenic mice showed partly reduced morphine withdrawal syndrome and decreased antinociception tolerance [20]. The latter effect was confirmed with the use of AC-263093, which activates only NPFFR2 [19,20]. Nevertheless, the involvement of the NPFFR1 receptor in attenuating morphine withdrawal syndrome was also shown in a later article [21].

Intravenous (iv) or icv pre-treatment with NPFF reduces the cessation of breathing manifested by apnea, and limits hypotensive and bradycardic action induced by endomorphin-1, an endogenous agonist of μ opioid receptors. These anti-opioid effects were more pronounced after icv pre-treatment with NPFF and were abrogated after NPFF receptor blockade [18]. NPFF alone does not affect breathing but, when administered either peripherally or centrally, it causes an increase in blood pressure and tachycardia [18,45], the opposite acute effects to opioids.

Continued work is underway to develop hybrid compounds consisting of opioid and neuropeptide FF pharmacophores characterized by limited opioid side effects. One of them, DN-9, which is given intrathecally and has been tested in various pain models, produced potent antinociception without tolerance; furthermore, it produced limited additional side effects typical of opioids, such as gastrointestinal transit inhibition, locomotor dysfunction, and hypotension [46].

## 3. Oxytocin (OT)

Oxytocin is a peptide hormone produced in the hypothalamus and released by the posterior pituitary gland into the bloodstream. Its main biological function is modulating social bonding, reproduction, childbirth and lactation, through activation of the Gq-protein-coupled oxytocin receptor [47].

Oxytocin has also been shown to affect pain sensitivity. Administration of OT, or brain stimulation facilitating its release, increases pain tolerance and diminishes acute pain both in humans and rodents [22]. An antinociceptive effect can be the result of presynaptic activation of dorsal horn OT receptors exciting GABAergic interneurons that, in a presynaptic way, inhibit Aδ- and C-fiber signals at nociceptive neurons [22]. Another indirect way to induce analgesia by OT is to activate the opioid system by evoking the release of endogenous opioids—such as Leu- and Met-enkephalins and β-endorphins—in the periaqueductal gray, the neural structure regulating the pain process [23].

OT has no direct agonistic effect on opioid receptors, yet treatment with OT increases opioid receptor signalling induced by various opioids. Furthermore, antinociceptive OT activity can be partially antagonized by opioid receptor blockers. Therefore, it is suggested that OT may function as a positive allosteric modulator, to induce an analgesic effect by enhancing the efficiency of opioid receptor signalling [24].

Oxytocin has been shown to regulate cardiovascular and respiratory functions, and its effect on both is definitely stimulatory. OT microinjected in the nucleus tractus solitarii (NTS) produces increases in blood pressure and heart rate due to OT receptor stimulation [48,49].

An excitatory effect on respiration was evoked after an iontophoretic OT application on NTS respiratory neurons [50]. Moreover, OT released from a stimulated hypothalamic paraventricular nucleus was able to enhance respiratory drive to respiratory muscles via oxytocin receptors in the pre-Bötzinger complex (the generator of respiratory rhythm), as well as augmenting arterial blood pressure and heart rate [51]. Recently, a human study by Jain et al., [52] reported that intranasal administration of oxytocin increases respiratory rate and reduces the duration and/or frequency of obstructive events, oxygen desaturation, and incidence of bradycardia in obstructive sleep apnoea patients. Returning to the context of anti-opioid activity, the latest findings by Brackley and Toney, [25] demonstrated that post-opioid cardiorespiratory depression can be reversed by oxytocin receptor activation. Systemic OT was highly effective, in a dose-dependent fashion, in reversing fentanyl-induced cardiorespiratory depression in rats; this suggests that selective stimulation of the OT receptor has therapeutic potential in rescues from opioid overdose.

## 4. Tyr-MIF-1 Family Peptides

The Tyr-MIF-1 family consists of at least four small peptides exhibiting heterogeneous properties and binding sites (Table 2). Despite similarities in structure, peptides of this family present different affinities for their own and opioid receptors, and therefore may act differently to modify opioid or anti-opioid effects. One of the peptides is melanocyte-inhibiting factor (MIF-1), the C-terminal tripeptide of oxytocin (Pro-Leu-Gly-NH2) that reduces the analgesic activity of morphine [53,54,55] and blocks morphine induced development of tolerance and dependence [54]. Receptor binding sites have not been identified for MIF-1, but it is known not to bind to the Tyr-MIF-1 binding site or to opioid receptors [55].

In contrast to MIF-1, the other peptides in the Tyr-MIF family, Tyr-MIF-1 and Tyr-W-MIF-1, show affinity not only to their own binding sites but also towards mu opioid receptors [64]. Tyr-MIF-1 shows mostly anti-opioid activity; it significantly decreases the analgesic effect of morphine in acute pain models [59], and impairs the development of morphine antinociceptive tolerance [61]. Tyr-MIF-1 has also been displayed to exacerbate abstinence syndrome in morphine-dependent rats [55].

A second peptide with affinity for its own and mu receptors is Tyr-W-MIF-1. When administered icv, it can antagonize morphine- and DAMGO-induced analgesia, most likely through blockade of the supraspinal mu 1 receptor [56]. However, it also shows opioid-like activity and, when administered centrally, it can induce naloxone-reversible analgesia in rats, occurring mostly via stimulation of mu 2 receptor [56,57]. It has also been demonstrated that rats pretreated with Tyr-W-MIF-1 exhibited tolerance to morphine analgesia [62].

The last member of the family that does not bind to opioid receptors, Tyr-K-MIF-1, inhibits the analgesic effect of morphine and induces antinociception through histamine release and stimulation of postsynaptic H1- and H2-receptors [55,58].

The effects of all of these peptides on opioid-induced respiratory and cardiovascular effects have not been studied extensively. Unlike naloxone, MIF-1 does not antagonize the elevations of heart rate and blood pressure produced by intravenous Leu5-enkephalin [65]. It was also displayed that MIF-1, at high doses, has blood pressure-lowering effects in experimental animals [66,67].

## 5. Ghrelin

Ghrelin is a growth-hormone-releasing peptide and an endogenous ligand for the growth hormone secretagogue receptor GHS-R1a, a metabotropic Gq protein-coupled receptor. Ghrelin is involved in a broad range of physiological effects, including regulation of appetite, metabolism, the heart, the nervous system, endocrine and exocrine function, and the immune and reproductive systems [68,69,70]. Ghrelin has also been found to play a role in regulating pain perception by interacting with the opioid system. Centrally given, it reduces inflammatory hyperalgesia [71], acute pain [26], and visceral sensation [72], effects that were attenuated by naloxone treatment. According to Liu et al., [27], icv-applied ghrelin activated the GHS-R1a, which increased the release of the endogenous proenkephalin peptide, which produced antinociception via δ-opioid receptor stimulation. Interaction of GHS-R1a with the central opioid system was previously reported in ghrelin and ghrelin(1–7)-NH2 produced antinociception in an acute model of pain [26,73]. The pro-opioid effects of ghrelin were demonstrated by administration of a ghrelin receptor agonist (HRRP-2, hexarelin), which enhanced the analgesic effect induced by morphine [74,75]. One study showed the opposite anti-opioid effect of ghrelin, which was able to inhibit systemic morphine-induced analgesia, not blocked with the GHS-R1a antagonist [70].

There is little information on the impact of ghrelin and its derivatives on tolerance to morphine. One finding by Baser et al. [74] suggests that hexarelin, in combination with morphine, attenuates analgesic tolerance to morphine. In another study, obestatin, which is ghrelin-associated peptide derived from preproghrelin, attenuated analgesic tolerance to morphine and reversed the effect of morphine withdrawal in mice [76]. The total picture that emerges from the above studies is a pro-opioid effect of ghrelin, enhancing the morphine analgesic effect, which depends on the interaction of GHS-R1a and opioid receptors. On the subject of addiction, icv ghrelin has been demonstrated to increase heroin consumption and seeking [77]. Interestingly, intraperitoneal antagonist of the ghrelin receptor, JMV2959, diminished morphine-evoked rewarding properties and behavioral stimulation [78,79].

Studies on the effects of ghrelin on post-opioid respiratory depression and cardiovascular changes are lacking. However, it is known that ghrelin alone induces vasodilatation, leading to a decrease in mean arterial pressure without changing the heart rate [68].

## 6. Cholecystokinin (CCK)

CCK plays an important physiological role as a peptide hormone in the gut that stimulates the release of enzymes responsible for fat and protein digestion. There are several isoforms of CCK differing in the number of amino acids, such as CCK58, CCK33, CCK22, and CCK8. In the brain, where the prevailing form of CCK is the sulfated octapeptide, CCK-8, it regulates satiety, anxiety, but also cognition, reward, learning and memory [9,17]. CCK receptors are a group of G-protein coupled receptors; CCK-A receptor, distributed primarily in the gastrointestinal tract, and CCK-B, mostly in the brain [80]. Both sulfated and non-sulfated CCK-8 are potent at CCK-B receptors, whereas only sulfated CCK-8 shows affinity towards CCK-A receptors, but much less than for CCK-B receptors [81].

CCK-8 has been proposed to be an anti-opioid peptide and, in fact, administered systemically or centrally, antagonizes the effect of analgesia produced by morphine and β-endorphin [28,29,30], most probably acting via a CCK-B receptor [31,32]. The latter was confirmed by studies with L-365,260 treatment, a specific antagonist of the cholecystokinin type B receptor, which enhanced opioid-evoked analgesia and blocked CCK-induced inhibition of opioid analgesia [31,82]. A recently proposed molecular mechanism for CCK-8 antagonism to opioid analgesia may involve heteromerization of opioid and CCK-B receptors, which reduces opioid receptor activity consisting of reduced ligand binding affinity and agonist-induced ERK1/2 phosphorylation [32]. This corresponds with a previous study using mice deprived of the CCK-B receptor, in which a lack of negative feedback control from CCK-B receptors led to upregulation of the opioid system, such as spontaneous hyperlocomotion, hyperalgesia and more severe withdrawal syndromes after chronic morphine treatment [83]. The authors explained the hyperalgesia phenomenon as a small increase in endogenous opioids leading to activation of the NMDA-dependent pronociceptive system, resulting in hypersensitivity to pain that was reversed by treatment with an NMDA antagonist [83].

It appears that CCK may also exert an analgesic effect, but this is postulated to be related to higher doses of the peptide and selective stimulation of the CCK-A receptor, possibly leading to the release of endogenous enkephalins [84,85,86].

Blockade of the CCK system appears to be involved in the inhibition of opioid tolerance, as morphine tolerance was reduced by treatment with antagonists of both CCK receptor subtypes; however, a greater role for the CCK-B receptor has been indicated [9,82,87,88].

There are no data on whether CCK affects post-opioid respiratory depression. The only study to analyze the effect of CCK-B receptor blockade with devazepide found that it had no effect on morphine-induced respiratory depression while enhancing its analgesic effects [89].

The respiratory and circulatory effects of CCK alone are difficult to clearly categorize. CCK-8, applied centrally or systemically, has been observed to evoke various respiratory responses: stimulation [90,91], but also short-lived respiratory depression [92], even with episodes of prolonged apnea after the icv challenge [93]. Regarding the effects on blood pressure, both hypotensive and pressor effects have been observed [92].

## 7. Nociceptin/Orphanin FQ (N/OFQ)

Nociceptin is a peptide related to the opioid class of compounds, widely distributed in the central nervous system [11]. Although it does not act on the classical opioid receptors (μ, κ, and δ), it does have affinity for the opioid receptor-like-1 (ORL1). ORL1, which shows 60% homology overall with opioid receptors, is thought to belong, structurally, to the family of opioid receptors. Contrarily to classic opioid receptors, its action is not sensitive to naloxone [94]. N/OFQ is engaged in the pain sensation, but its effect can be manifold. Injected in the brain, it produces hyperalgesia [33,95], but administered to the spinal cord, it evokes an analgesic response [96,97].

N/OFQ administered icv acts like an anti-opioid peptide to counteract the analgesic effects of the endo- or exogenous opioids [33,34]; conversely, blockade of its signaling leads to an increased pain threshold, antinociception, and potentiation of the analgesic effects of opioids with a likely diminution in opioid-induced tolerance [94,98,99]. Colocalization of nociceptin receptors with μ-opioid receptors in many brain regions, and the fact that both share common signaling pathways, may result in crosstalk between these receptors; furthermore, this may result in the possibility of counteracting the side effects of μ-opioid peptide receptor stimulation by simultaneous action on N/OFQ receptors [99]. An example of a synthetic drug that simultaneously acts at opioid and N/OFQ receptors is cebranopadol, which displays reduced respiratory depression [100]. Cebranopadol showed a larger therapeutic window between antinociception and respiratory depression than fentanyl in rats [100], and prevented the onset of full respiratory depression occurring after opioid-receptor-only activation in humans [101]. Cebranopadol has been successful in phase III clinical trials, being safe and well tolerated, and as effective as morphine in patients with chronic-cancer-related pain [102,103].

Regarding the respiratory effects of nociceptin itself, its role in breathing control remains unclear; however inspiratory rhythm slowing has been described in an in vitro brainstem preparation of newborn rats, most likely via its direct effects on the pre-Bötzinger complex (respiratory rhythm generator) [104]. The effect of N/OFQ on blood pressure after systemic administration was definitely hypotensive in both anaesthetized rats [105,106] and conscious mice [107]. However, central administration showed the opposite effects of rostral NTS-dose-dependent increase in arterial blood pressure and heart rate in conscious rats [108], and commissural NTS-depressor and bradycardic responses in anesthetized rats [109].

## 8. Endothelin

There has been increased interest in the endothelin (ET) peptide, whose physiological role is to influence vascular tone and blood pressure [110]. In 2002, for the first time, the potentiating effect of central endothelin A receptor antagonism, ET_A_, on morphine-induced analgesia and hyperthermia was demonstrated in rats [35]. It was also later shown in mice that the same ET_A_ receptor antagonist, BQ123—which does not act on opioid receptors—when administered icv, alleviates opioid-induced withdrawal symptoms [111]. Recently, a novel selective ET_A_ receptor antagonist, named Compound E, has been shown to increase the morphine withdrawal threshold and analgesic effect; this is likely mediated by dimerization of ET_A_ and opioid receptors [112]. An additional advantage of this compound, if it were to be considered in the category of a potential drug, is its action after oral administration, indicating that it crosses the blood brain barrier.

## 9. Venom Peptides

An interesting and huge group of peptides with potential pro-opioid effects, that still require further research on their properties, are venom peptides.

Phα1β is a peptide from the venom of the spider *Phoneutria nigriventer*, which induces analgesic effect in rodent models of chronic and acute pain via blockade of neuronal voltage sensitive calcium channels; this prevents calcium influx, depolarization and neurotransmitter release from central and peripheral nerves [60]. Its intrathecal or intravenous injection exerts prolonged analgesia without significant side effects in mice and rats [60,63].

Furthermore, intrathecal Phα1β potentiates morphine-induced analgesia in mouse models of thermal, mechanical and postoperative pain, while reducing opioid side effects such as tolerance and withdrawal syndrome [36,37]. Phα1β and methadone, when applied in combination, presented synergistic interaction and exert potentiation of analgesia in a mouse model of cancer pain, without motor function adverse effects, and restoring morphine-induced tolerance [113].

Another peptide exhibiting an opioid regulatory function by blocking voltage-gated calcium channels is lecontide, which is a venom peptide belonging to the ω-conotoxin, isolated from the venom of the marine cone snail. Although it evoked a weak antihyperalgesic effect by itself, lecontide, when applied iv in combination with intraperitoneal morphine, increased its reversal effect on hyperalgesia in an animal model of bone cancer [114]. Ziconotide (MVIIA, SNX111), a synthetic form of ω-conotoxin derived from the *Conus magnus* toxin, is a highly potent analgesic when administered intrathecally that does not induce drug addiction or tolerance like morphine [115]; however, when injected iv, it produces cardiovascular side effects such as tachycardia and orthostatic hypotension [116].

Promising peptides not yet completely studied are those identified in tarantula venom, μ-theraphotoxin-Pn3a (μ-TRTX-Pn3a) or phlotoxin-1 (PhlTx1) [117,118,119]. Both are selective inhibitors of the voltage-gated sodium channel Na_V_1.7; their role as antinociceptive targets was identified when a loss of function mutation in the gene encoding Na_V_1.7 led to a congenital inability to sense pain [117]. It is interesting to note that these peptides do not exhibit antinociceptive effects on their own, and only when administered together with exogenous opioids do they induce analgesia, allowing the opioid dose to be significantly reduced. [117]. The mechanism of this synergistic effect of opioid receptor agonists with selective Nav1.7 inhibitors is not yet known; nevertheless, this may represent a new approach in pain management.

## 10. Conclusions

Potent opioid analgesics remain the mainstay of therapy for the relief of moderate to severe acute nociceptive pain. Due to opioid-related side effects such as respiratory depression, tolerance, and dependence, further research and the search for compounds that counteract them is needed. The opioid-modulating peptides described above provide such opportunities, and the use of their agonists or antagonists can spare or even enhance the analgesic effect of opioids, while reducing opioid-induced respiratory depression. The administration of opioids, in combination with agonists and antagonists of these peptides, may reduce the amount of opioid needed for analgesia and, thus, also allow for a reduction in its unwanted effects. Caution is needed, however, because some of the peptides may themselves cause side effects, such as respiratory depression or hypotension. It is promising that novel compounds acting on specific receptors, which include BN-9, cebranopadol, or Compound E, as described in the article, are being developed to potentiate the analgesic effects of opioids while minimizing their side effects.

## Figures and Tables

**Table 1:** Summary of the main pro- and anti-opioid effects of the described peptides. For more detailed information see the text.

Peptide (Sequence)	Pro-Opioid Activity	Anti-Opioid Activity	Receptors
NPFF (FLFQPQRFa)	➢ (it) potentiation of opioid antinociception [15]	➢ (icv, supraspinal) reversal of opioid-induced analgesia [16,17]	NPFF [15,18]
		➢ (icv) elimination of opioid-induced apnea and reduction in EM-1-induced hypotension and bradycardia [18]	NPFF [15,18]
		➢ reduction in morphine tolerance [19,20]	NPFF2 [19,20]
		➢ reduction in morphine withdrawal syndrome [20,21]	NPFF1, NPFF2 [20,21]
Oxytocin (CYIQNCPLG)	➢ antinociceptive effect [22,23]		OT and opioid receptors [22,24]
		➢ reversal of post-opioid cardio-respiratory depression [25]	OT receptors [25]
Ghrelin (GSSFSPEHQKAQQRKESKKPPAKLQPR)	➢ enhancement of morphine analgesic effect [26,27]		interaction of GHS-R1a and opioid receptors [27]
CCK-8 (DXMGWMDF)		➢ (iv, icv) antagonism of opioid-induced analgesia [28,29,30]	CCK-B [31,32]
Nociceptin/ orphanin FQ (H-FGGFTGARKSARKLANQ-OH)		➢ (icv) antagonism of opioid-induced analgesia [33,34]	ORL1 [34]
Endothelin (CSCSSLMDKECVYFCHLDIIW)		➢ blockade of ET_A_ receptor, enhanced morphine-induced analgesia and hyperthermia [35]	ET_A_ [35]
Phα1β-venom peptide	➢ potentiates morphine-induced analgesia [36,37]	➢ reduction in morphine tolerance and withdrawal syndrome [36,37]	

**Table 2:** Tyr-MIF-1 family peptides; binding sites and impact on different morphine (MF) effects.

	Peptide (Sequence)	MIF-1 (PLG)	Tyr-MIF-1 (YPLG)	Tyr-W-MIF-1 (YPWG)	Tyr-K-MIF-1 (YPKG)
Binding sites [55]	Tyr-K-MIF-1	−	−	−	+
	Tyr-MIF-1	−	+	+	+
	MOR	−	+	+	−
	DOR	−	−	−	−
	KOR	−	−	−	−
	Opiate effects		+	+	+
	Antiopiate effects	+	+	+ (mu1) [56]	+
	Analgesia induction		+	+ (mu2) [56,57]	+ (histaminergic system) [58]
Effects on	MF analgesia	antagonism [55]	decrease [59]	antagonism [56,59]	decrease [55]
	MF tolerance	blockade [60]	decrease [61]	increase [62]	
	MF dependence	blockade [60]			
	MF abstinence syndrome		increase [63]		
